# Comparative Epidemiology of Human Infections with Middle East Respiratory Syndrome and Severe Acute Respiratory Syndrome Coronaviruses among Healthcare Personnel

**DOI:** 10.1371/journal.pone.0149988

**Published:** 2016-03-01

**Authors:** Shelan Liu, Ta-Chien Chan, Yu-Tseng Chu, Joseph Tsung-Shu Wu, Xingyi Geng, Na Zhao, Wei Cheng, Enfu Chen, Chwan-Chuen King

**Affiliations:** 1 Department of Infectious Diseases, Zhejiang Provincial Centre for Disease Control and Prevention, Hangzhou, Zhejiang Province, China; 2 Center for Geographic Information Science, Research Center for Humanities and Social Science, Academia Sinica, Taipei, Taiwan; 3 Institute of Epidemiology and Preventive Medicine, College of Public Health, National Taiwan University, Taipei, Taiwan; 4 Institute of Statistical Science, Academia Sinica, Taipei, Taiwan; 5 Luke International, Malawi Office and Mzuzu University, Mzuzu City, Malawi; 6 Emergency Offices, Jinan Centre for Disease Control and Prevention, Jinan, Shandong Province, China; 7 National Research Center for Wildlife Born Diseases, Key Lab of Animal Ecology and Conservation Biology, Institute of Zoology, Chinese Academy of Sciences, Beijing, China; The Chinese University of Hong Kong, HONG KONG

## Abstract

The largest nosocomial outbreak of Middle East respiratory syndrome (MERS) occurred in South Korea in 2015. Health Care Personnel (HCP) are at high risk of acquiring MERS-Coronavirus (MERS-CoV) infections, similar to the severe acute respiratory syndrome (SARS)-Coronavirus (SARS-CoV) infections first identified in 2003. This study described the similarities and differences in epidemiological and clinical characteristics of 183 confirmed global MERS cases and 98 SARS cases in Taiwan associated with HCP. The epidemiological findings showed that the mean age of MERS-HCP and total MERS cases were 40 (24~74) and 49 (2~90) years, respectively, much older than those in SARS [SARS-HCP: 35 (21~68) years, *p =* 0.006; total SARS: 42 (0~94) years, *p =* 0.0002]. The case fatality rates (CFR) was much lower in MERS-HCP [7.03% (9/128)] or SARS-HCP [12.24% (12/98)] than the MERS-non-HCP [36.96% (34/92), *p<0*.*001*] or SARS-non-HCP [24.50% (61/249), *p<0*.*001*], however, no difference was found between MERS-HCP and SARS-HCP [*p = 0*.*181*]. In terms of clinical period, the days from onset to death [13 (4~17) vs 14.5 (0~52), *p* = 0.045] and to discharge [11 (5~24) vs 24 (0~74), *p =* 0.010] and be hospitalized days [9.5 (3~22) vs 22 (0~69), *p* = 0.040] were much shorter in MERS-HCP than SARS-HCP. Similarly, days from onset to confirmation were shorter in MERS-HCP than MERS-non-HCP [6 (1~14) vs 10 (1~21), *p =* 0.044]. In conclusion, the severity of MERS-HCP and SARS-HCP was lower than that of MERS-non-HCP and SARS-non-HCP due to younger age and early confirmation in HCP groups. However, no statistical difference was found in MERS-HCP and SARS-HCP. Thus, prevention of nosocomial infections involving both novel Coronavirus is crucially important to protect HCP.

## Introduction

Ten years after the outbreaks of Severe Acute Respiratory Syndrome (SARS) occurred in 29 countries, another novel coronavirus named Middle East Respiratory Syndrome coronavirus (MERS-CoV) was first found in the Middle East, in 2012 [1, 2]. The novel coronaviruses responsible for both SARS and MERS infections are zoonotic viruses. Civet cats and bats were identified as the sources of SARS-CoV infections in humans, whereas camels and bats were associated with human MERS-CoV infections [3–5]. Both viruses spread to an intermediate mammalian host from which humans are infected. Although human-to-human transmission occurred, this was mainly within households and medical care facilities, where healthcare personnel (HCP) were affected the most. However, super spreading events were reported in the SARS and MERS outbreak [6, 7].

Previous studies also indicated that HCP were a major high-risk population for infections with SARS-CoV and MERS-CoV [8, 9], but the information was very fragmented. Here, based on a series of HCP laboratory-confirmed MERS and SARS infections obtained from the World Health Organization and Taiwan, respectively, we compared the epidemiological and clinical characteristics, and particular similarities as well as differences between the two coronavirus infections to measure the risks related to fatal outcomes in both HCP and total cases. These data will be of benefit for effective prevention and control for any future nosocomial outbreaks of novel Coronavirus, and will therefore promote occupational health.

## Materials and Methods

### Sources of study populations

All the laboratory-confirmed MERS cases were obtained from various publicly available sources from April 1 of 2012 to July 7 of 2015, including the published literature and data reported to the World Health Organization (http://www.who.int/csr/don/archive/disease/coronavirus_infections/en/) (**S1 Table)**. All laboratory-confirmed SARS cases were reported to the WHO from Taiwan during November 1 of 2002 to December 31, 2003 (http://www.who.int/csr/sars/country/table2004_04_21/en/), after the final decision by the SARS Advisory Committee members in Taiwan (**S2 Table)**.

### Case definitions

According to the WHO guideline, a laboratory-confirmed case with MERS-CoV was defined as as either detection of viral nucleic acid or confirmation by serology [10].Based on the WHO guideline, the case definitions for SARS cases were confirmed by several different laboratory tests, including confirmation of the RT-PCR results from two different laboratories or two different specimens collected from the same person, and/or serological tests by ELISA.

### Epidemiologic investigations and laboratory tests

Once cases were suspected to be clinical SARS cases, preliminary investigation was conducted using a standardized questionnaire about demographic characteristics and exposure history, clinical features, and laboratory results. Respiratory samples obtained from any reported suspected SARS cases were sent to the Center for Disease Control in Taiwan (Taiwan-CDC) for confirmation by RT-PCR or serology. The details of all the laboratory methods (molecular RT-PCR or serological tests) to confirm SARS-CoV were as previously described [11].

### Statistical analysis

Regional incidence rates are shown on the map using the bio-geographical information system software ArcGIS Map10.2 (http://resources.arcgis.com/en/home/). We used the ggplot function from the R package “ggplot2” to draw the boxplots of age distribution between MERS and SARS cases.

Quantitative measurements are presented as the mean and range of the observed values, and qualitative measurements are presented as relative and absolute frequencies. Categorical and continuous characteristics were analyzed with a chi-squared test and t test, respectively. All statistical analyses were conducted using SAS 9.2 (SAS Institute, Cary, NC, USA). Any *p* values given are two-sided and subject to a local significance level of 0.05.

## Results

### Epidemiological comparison

#### Geographical distribution

**Fig 1:**
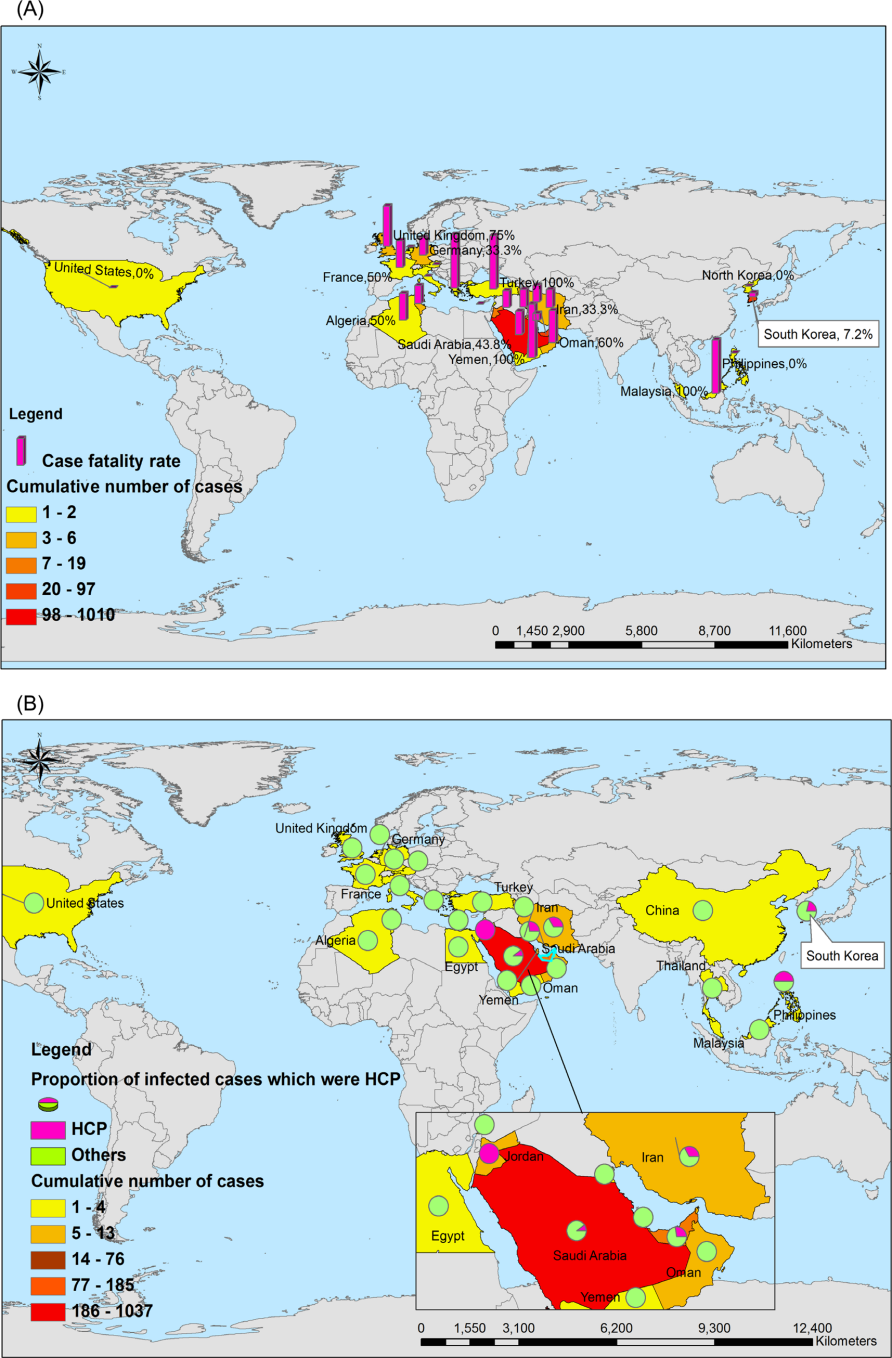
Geographical distribution of 1368 laboratory-confirmed cases, 487 deaths and 183 healthcare personnel (HCP) infected with MERS-CoV from April 1 of 2012 to July 7 of 2015. Notes: 1a: Overall MERS cases and the case fatality rate. The denominator is the cumulative number of total cases with MERS-CoV; Numerator is the case fatality rate in total cases. 1b: Overall MERS cases and percent of HCP with MERS infections. The denominator is the cumulative number of total cases with MERS-CoV; Pie represents the percentage of HCP and non HCP among the total cases.

As of 7 July 2015, 1368 laboratory-confirmed human MERS-CoV cases had been reported to the WHO, 487 of whom died (35.6%). All of these MERS cases were distributed in 26 countries, particularly predominant in the Arabian Peninsula and South Korea. However, global MERS-HCP cases occurred in 6 countries; see Table 1 **and Fig 1.** MER-HCP accounted for 13.37% (183/1368) of overall MERS cases in the world.

**Table 1:** Epidemiologic comparison of the laboratory-confirmed human infections with MERS-CoV and SARS-CoV* in healthcare personnel (HCP) and total cases as of July 7, 2015.

Epidemiologic Characteristics	MERS-CoV (+)			SARS-CoV (+)			Comparisons	
	HCP (n = 183)	Total (n = 1368)	*p*_*1*_	HCP (n = 98)*	Total (n = 8096)	*p*_*2*_	*p*_*3*_	*p*_*4*_
**Outbreak Area**								
No. of countries/areas	6	26	NA	9	29	NA	NA	NA
Most affected Countries	Saudi Arabia	Saudi Arabia	NA	China	China	NA	NA	NA
**Epidemic Curve**								
Starting month	April of 2012	April of 2012	NA	January of 2003	November of 2002	NA	NA	NA
Peaking month	Spring	Spring and summer	NA	NA	Spring and winter	NA	NA	NA
Months of epidemic period	>39	>39	NA	8	8	NA	NA	NA
**Clusters**								
Super-spreader	Yes	Yes	NA	Yes	Yes	NA	NA	NA
Cluster size	NA	2.3 (1~26)	NA	NA	8 (0~125)	NA	NA	NA
Outbreak areas	Hospitals (Hosp)	Hosp, Households (HH)	NA	Hospitals	Hosp, HH, Communities	NA	NA	NA
**Animal Sources**	No	Bat and camel	No	NA	Civet cat and bat	NA	NA	NA
**Human Cases**								
Percent of HCP cases	NA	13.37% (183/1368)	NA	NA	21.07% (1706/8096)	NA	0.000	NA
Mean age (yr)	40 (24~74)	49 (2~90)	0.035	35 (21~68)*	42 (0~94)	0.044	0.006	0.0002
Male/total%	54.46% (55/101)	65% (883/1359)	0.002	17.35% (17/98)*	46.93% (3778/8050)	0.027	<0.001	<0.001
Overall CFR	NA	35.6% (487/1368)	NA	NA	9.6% (774/8096)	NA	NA	<0.001
**Working place for HCP**								
Emergency rooms	17.65% (3/17)	NA	NA	31.58%（18/57)	NA	NA	0.943	NA
Intensive care units	58.82% (10/17)	NA	NA	10.53%（6/57)	NA	NA	<0.001	NA
Internal medicine wards	11.76% (2/17)	NA	NA	26.31% (15/57）	NA	NA	0.704	NA
Others	11.76% (2/17)	NA	NA	31.58%（18/57)	NA	NA	0.507	NA
Unknown	166	NA	NA	41	NA	NA	NA	NA

**Denotations: *p***_***1***_: comparison of HCP and non-HCP cases infected with MERS-CoV; ***p***_**2:**_ comparison of HCP and non-HCP cases infected with SARS-CoV; ***p***_***3*:**_ comparison of HCP infected with MERS-CoV and SARS-CoV; ***p***_***4***_**:** comparison of total MERS-CoV and total SARS-CoV cases. CFR: Case Fatality Rate; NA: No available data

*SARS-CoV Data were obtained from Taiwan.

**Fig 2:**
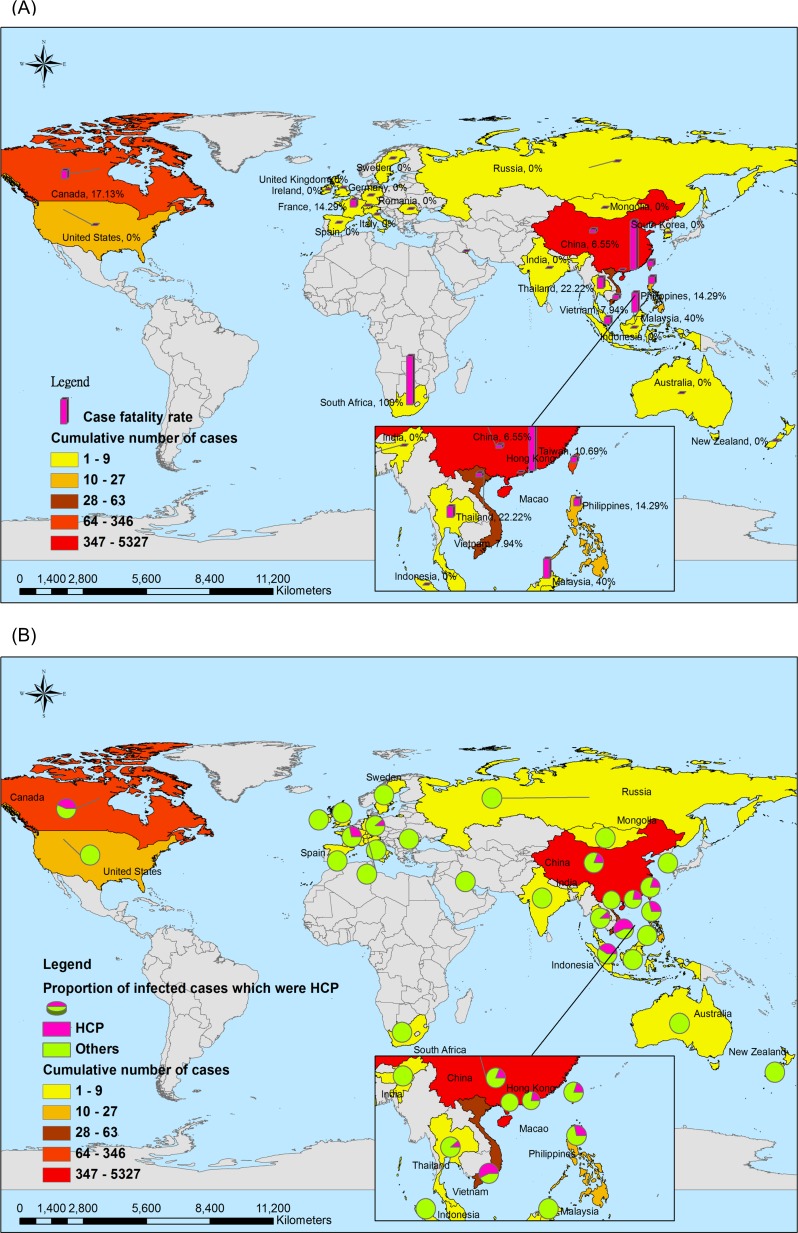
Geographical distribution of 8096 laboratory-confirmed cases, 774 deaths and 1706 HCP infected with SARS-CoV from November 1 of 2002 to December 31, 2003. Notes: 1a: Overall SARS cases and the case fatality rate. The denominator is the cumulative number of total cases with SARS-CoV; Numerator is the case fatality rate in total cases. 1b: Overall SARS cases and percent of HCP with SARS infections. The denominator is the cumulative number of total cases with SARS-CoV; Pie represents the percentage of HCP and non HCP among the total cases.

By contrast, as of 31 December 2003, 8096 laboratory-confirmed global human SARS-CoV cases had been reported to the WHO, 774 of whom died (9.6%). These SARS cases were distributed in 29 countries, particularly predominant in Asian areas. The percent of the SARS-HCP in total cases was higher than the percent of the MERS-HCP [21.07% (1706/8096) vs 13.37% (183/1368), *p*<0.001]. The CFR in overall MERS was statistically significantly higher than that of overall SARS [35.6% (487/1368) vs 9.6% (774/8096), *p*<0.001]. (**Table 1 and Fig 2).**

#### Demographical and occupational exposure

**Fig 3:**
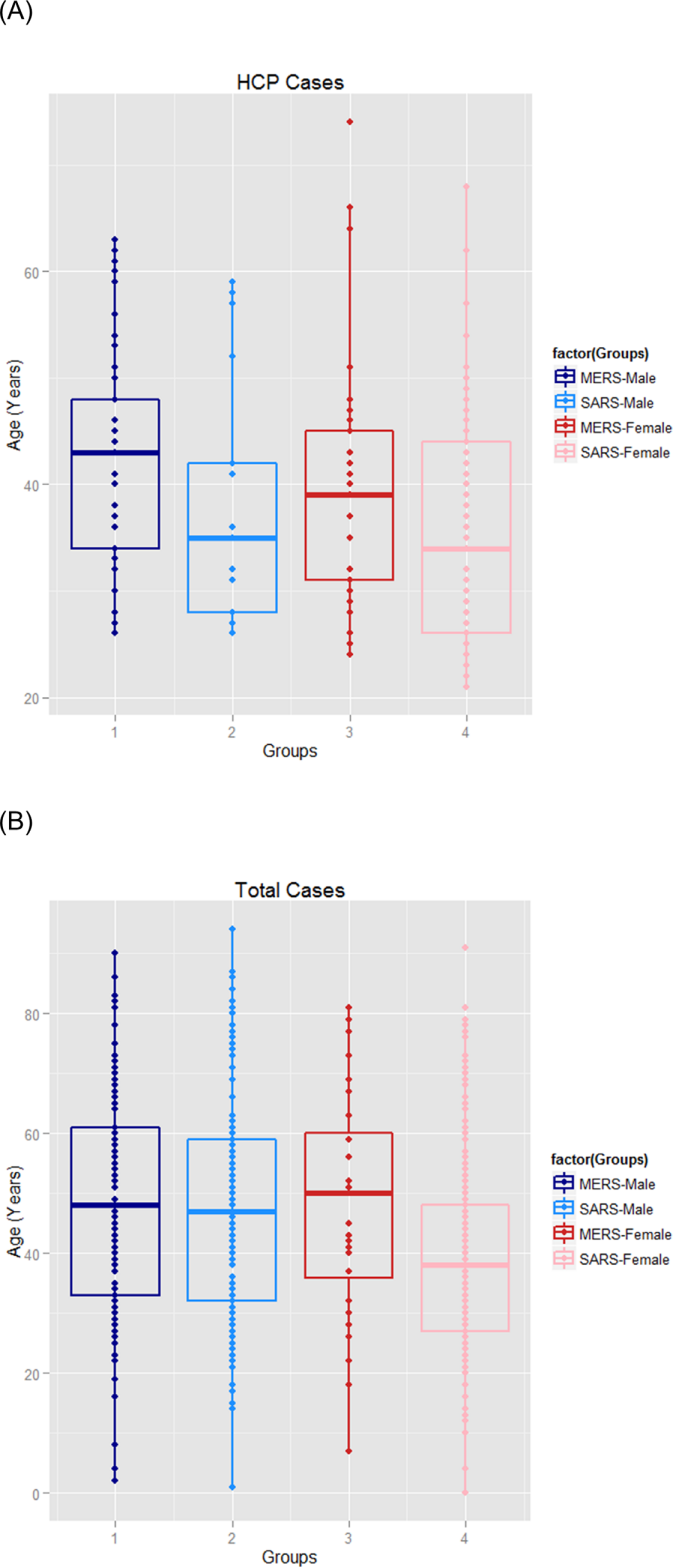
The age distribution in the laboratory-confirmed cases with MERS and SARS from Taiwan in overall population and HCP. Note: 3a: MERS (N = 174) and SARS (N = 347) infections in overall cases. 3b: MERS-HCP (N = 143) and SARS-HCP (N = 98) cases.

The mean age in worldwide total 174 MERS cases was 49 years (range: 2~90 years), of which 143 MERS-HCP cases were aged 40 years (range: 24~74 years), significantly younger than in 31 MERS-non-HCP cases [47.5 years (range: 2~90 years), *p* = 0.035]. Similarly, the mean age in 347 total Taiwan SARS cases was 42 years (range: 0~94), of which the mean age in 98 SARS-HCP cases was 35 years (range: 21~68 years), also much younger than that of SARS-non-HCP [45 years (range: 0~94 years), *p =* 0.044]. Noticeably, the mean ages of MERS-HCP and total MERS cases were significantly older than those for SARS-HCP (*p =* 0.006) and total SARS cases in Taiwan (*p =* 0.0002). (**Fig 3A and 3B and Table 1).** We stratified the age groups between the MERS-HCP and SARS-HCP cases; the results show that there are no signature differences in the mean age of the 20~, 30~, 40~, 50~, 60–70 groups between the two groups.

In terms of sex distribution, among MERS cases whose gender information was available, 65% of 1359 total MERS cases were male, of which 54.46% of 101 MERS-HCP and 69.55% of 1258 MERS-non-HCP [*p =* 0.002] were male respectively. However, in Taiwan females were dominant in the 98 SARS-HCP cases (82.65%, 81/98) and 249 SARS-non-HCP cases (55.0%, 137/249) [p = 0.027], while males accounted for 46.93% (3778/8050) of total global SARS cases. **(Fig 3A and 3B and Table 1).** We stratified the gender groups between the MERS-HCP and SARS-HCP cases, with the results showing significant differences in the gender distribution of the 20~, 30~, 40~, 50~, 60–70 groups between the two groups.

Regarding the working departments of HCP, 58.82% (10/17) of MERS-HCP cases worked in an intensive care unit (ICU), while 17.65% (3/17), 11.76% (2/17), and 11.76% (2/17) worked in Emergency rooms (ER), internal medicine and other departments in hospitals, respectively. Among the 57 of 98 SARS-HCP cases with their information of working departments available, ER (31.58%, 18/57), wards of internal medicine (26.31%, 15/57), and ICU (10.53%, 6/57) and other wards (31.58%, 18/57) were the four major working sites **(Table 1).** Our statistics for department will be heavily biased because of the missing department information in the HCP infections with MERS and SARS-CoV.

**Fig 4:**
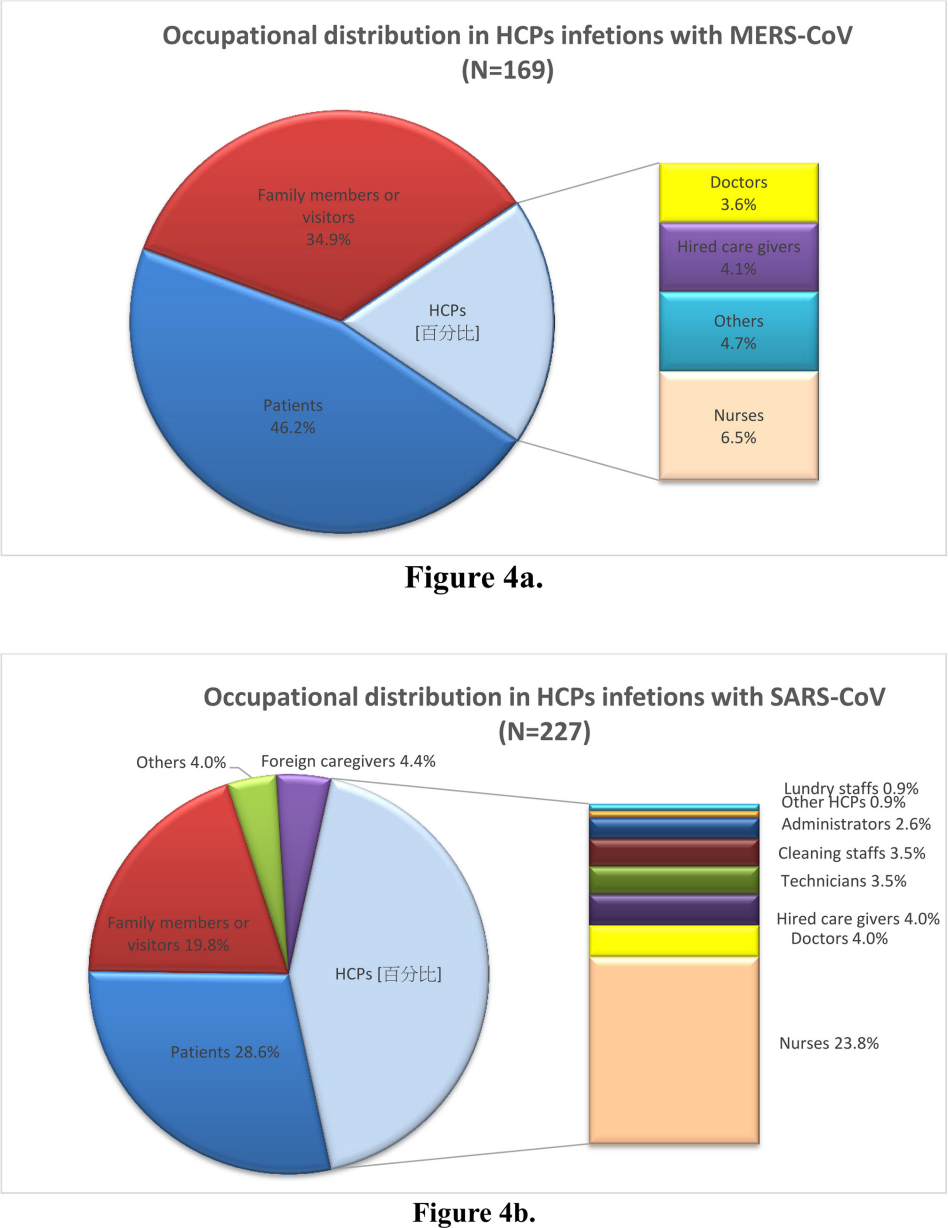
The occupational categories of hospital-associated MERS infections (n = 169) in Korea, 2015 and SARS infections (n = 227) in Taiwan, 2003. Notes: 4a: Occupational distribution in MERS-HCP. 4b: Occupational distribution in SARS-HCP.

Among total 169 hospital-acquired MERS cases, patients accounted for 46.2%, family members or visitors 34.9%, and HCP (19.0%)**.** Among the 227 laboratory-confirmed SARS cases from hospital infections, HCP accounted the highest (43.2%), followed by patients (28.6%), family members or visitors (19.8%), foreign caregivers (4.4%), and others (4.0%). Nurses were the major populations in both groups **(Fig 4A and 4B).**

### Clinical comparison

#### Clinical spectrum

The percentages of asymptomatic cases [36.72% (47/128) vs 17.39% (16/92), *p =* 0.001] and mild cases [43.75% (56/128) vs 18.48% (17/92), *p* <0.001] in HCP were much higher than those in non-HCP. However, the percentage of severe MERS cases in HCP was significantly lower than that in non-HCP [12.5% (16/128) vs 27.17% (25/92), *p =* 0.006)]. Similarly, CFR of MERS-HCP was statistically lower than that in MERS-non-HCP [(7.0% (9/128) vs 36.96% (34/92), *p*<0.001] **Table 2.** By contrast, severe SARS cases were more common in HCP than in non-HCP [86.76% (86/98) vs 75.50% (188/249), *p =* 0.012]. Nonetheless, CFR of SARS in HCP was significantly lower than that in non-HCP [12.24% (12/98) vs.24.50% (61/249), *p =* 0.012, **Table 2**]. In general, MERS-HCP were predominantly associated with mild cases whereas SARS-HCP were more likely to develop severe cases. On the other hand, MERS-non-HCP cases had higher CFR than SARS-non-HCP cases [36.96% (34/92) vs 24.50% (61/249), *p =* 0.023, **Table 2.**

**Table 2:** Clinical comparisons of the laboratory-confirmed human infections with MERS-CoV and SARS-CoV between healthcare personnel (HCP) and non-HCP.

Clinical Characteristics	MERS-CoV (+)			SARS-CoV (+)			Comparisons	
	HCP (n = 128)	Non-HCP (n = 92)	*p*_*1*_	HCP (n = 98) *	Non-HCP (n = 249)	*p*_*2*_	*p*_*3*_	*p*_*4*_
**Clinical Severity**								
Asymptomatic	36.72% (47/128)	17.39% (16/92)	<0.0001	0.00% (0/98)	0.00% (0/249)	0.012	<0.0001	<0.0001
Mild	43.75% (56/128)	18.48% (17/92)	<0.0001	0.00% (0/98)	0.00% (0/249)	0.012	<0.0001	<0.0001
Severe	12.50% (16/128)	27.17% (25/92)	<0.0001	86.76% (86/98)	75.50% (188/249)	0.012	<0.0001	<0.0001
Fatal	7.03% (9/128)	36.96% (34/92)	<0.0001	12.24% (12/98)	24.50% (61/249)	0.012	<0.0001	<0.0001
**Clinical Symptoms**								
Fever (>38°C)	58.54% (75/128)	97.83% (90/92)	<0.001	95.92% (94/98)	83.94% (209/249)	0.003	<0.001	<0.001
Cough	63.41% (81/128)	83.70% (77/92)	<0.001	33.67% (33/98)	38.55% (96/249)	0.397	<0.001	<0.001
Chills or rigors	55% (70/128)	84.78% (78/92)	<0.001	7.14% (7/98)	2.01% (5/249)	0.018	<0.001	<0.001
Sore throat	17.07% (22/128)	14.00% (13/92)	0.541	3.06% (3/98)	3.21% (8/249)	0.942	<0.001	<0.001
Runny nose	9.76% (12/128)	0.00% (0/92)	0.006	1.02% (1/98)	1.20% (3/249)	0.884	0.007	0.686
Haemoptysis	0.00% (0/128)	17.00% (16/92)	<0.001	0.00% (0/98)	0.00% (0/249)	NA	NA	NA
Shortness of breath	48.78% (62/128)	73.91% (68/92)	<0.001	1.02% (1/98)	8.43% (21/249)	0.010	<0.001	<0.001
Headache	0.00% (0/128)	9.78% (9/92)	<0.001	2.04% (2/98)	4.02% (10/249)	0.364	0.365	0.07
Myalgia	14.63% (19/128)	32.61% (30/92)	0.001	12.24% (12/98)	5.62% (14/249)	0.035	0.574	<0.001
Malaise	0.00% (0/128)	38.00% (35/92)	<0.001	1.02% (1/98)	1.20% (3/249)	0.884	0893	<0.001
Nausea	0.00% (0/128)	21.00% (19/92)	<0.001	1.02% (1/98)	0.00% (0/249)	0.628	0893	<0.001
Vomiting	0.00% (0/128)	21.00% (19/92)	<0.001	2.04% (2/98)	0.80% (2/249)	0.331	0.365	<0.001
Diarrhea	2.44% (3/128)	26.00% (24/92)	<0.001	13.27% (13/98)	12.05% (30/249)	0.757	0.004	0.002
Rhinorrhea	4.88% (6/128)	5.43% (5/92)	0.082	0.00% (0/98)	0.00% (0/249)	NA	0.079	0.002
**Disease Progression (Days)**								
Incubation period	7 (3~11)	5 (2.0~15)	0.150	6 (1~15)	5 (4~6)	0.250	0.890	0.640
From onset to admission	5 (0~11)	5 (1~14)	0.480	2 (0~14)	3 (0~42)	0.261	0.010	0.470
From onset to confirmation	6 (1~14)	10 (1~21)	0.044	2 (0~20)	3 (0~42)	0.416	0.250	0.770
From onset to death	13 (4~17)	12 (1~52)	0.930	14.5 (0~52)	12 (0~83)	0.277	0.045	0.200
From onset to be discharged	11 (5~24)	12 (3~30)	0.690	24 (0~74)	19 (2~92)	0.002	0.010	0.010

**Denotations:** *p*_*1*_: comparison of HCP and Non-HCP cases infected with MERS-CoV; *p*_*2*:_ comparison of HCP and Non-HCP cases infected with SARS-CoV; *p*_*3*_ comparison of HCP infected with MERS-CoV and SARS-CoV; *p*_*4*_ value represents comparison of Non-HCP infected with MERS-CoV and SARS-CoV. NA: No available data

*SARS-CoV Data were obtained from Taiwan.

#### Clinical manifestations

Detailed investigations on clinical manifestations of these two infections in HCP and non-HCP demonstrated that cough was the most frequent symptom in MERS-HCP [63.41% (81/128)], whereas fever was the most common symptom in MERS-non-HCP [97.83% (90/92)]. Additionally, both diarrhea and shortness of breath (SOB) were higher in the MERS-non-HCP compared to the MERS-HCP [26.00% (24/92) vs 2.44% (3/128), *p<*0.001; *73*.*91*% (68/92) vs 48.78% (62/128), *p<*0.001, respectively] (**Table 2**).

#### Time periods for clinical progress

Three time periods useful for public health surveillance were evaluated **(Table 2).** In MERS groups, the incubation period (the days from exposure to the clinical presentations) showed no differences between MERS-HCP and MERS-non-HCP [7 (3~11) vs 5 (2.0~15), *p =* 0.150] or between SARS-HCP and SARS-non-HCP cases [6 (1~15) vs 5 (4~6), *p =* 0.250]. The days from onset to being discharged and to death between the two groups also revealed similar results. By contrast, MERS-HCP cases had significantly shorter days from onset to laboratory-confirmation than MERS-non-HCP [6 (1~14) vs 10 (1~21), *p =* 0.044].

In SARS groups, among the three measures, only the days from onset to being discharged showed a difference, with significantly shorter days for SARS-HCP than SARS-non-HCP [19 (2~92) vs 24 (0–74), *p =* 0.002]. **Table 2**

Among the HCP with SARS and MERS infections, MERS-HCP had significantly longer days from onset to admission than SARS-HCP [5 (0~11) vs 3 (0~14), *p =* 0.010] but a shorter period from onset to being discharged [11 (5~24) vs 24 (0~74), *p =* 0.010)] and to death [13 (4~17) vs 14.5 (0~52), *p =* 0.045] and be hospitalized days [9.5 (3~22) vs 22 (0~69), p = 0.040] in MERS-HCP than those in SARS-HCP. **Table 2**

To minimize the case fatality rate in MERS-HCP and SARS-HCP, we analyzed the epidemiology about the deaths related to HCP infections. 10 fatal MERS-HCP cases from Saudi Arabia, UAE and Jordan, as well as 13 fatal SARS-HCP cases in Taiwan were included **(Table 3).** The mean age of fatal MERS-HCP cases was 50 (26~67), 70% of them were male, and 40% (4/10) had comorbidities. Similarly, the mean age of the fatal SARS-HCP cases was 49 (28~68), 64.29% of whom were female. 69.23% (9/13) of SARS-HCP cases had accompanying co-morbidities. We found that fatality was most often found in age 50~ groups with co-mortality in both the MERS-HCP and SARS-HCP groups respectively. Most of the fatal SARS-HCP were hired care workers (30.77% (; 4/13), or cleaners or nurses (15.38% (; 2/13). Importantly, the mean age in fatal HCP with the two infections was much older than in survivors of HCP with these infections.

**Table 3:** Clinical and epidemiological characteristics of fatal infections with MERS-CoV vs SARS-CoV in healthcare personnel (HCP).

Groups	Epidemiological information						Clinical information			Data source
	Countries	Case type	Age	Gender	Occupation	Working place	Comorbidity	Outcome	Death Dates	
HCP-MERS	Saudi Arabia	Confirmed	41	F	HCP	Unknown	No	Deceased	Unknown	http://www.moh.gov.sa/en/CCC/pressreleases/pages/mediastatement-2013-09-05-001.aspx
HCP-MERS	Saudi Arabia	Confirmed	56	Unknown	HCP	Unknown	No	Deceased	Unknown	http://www.moh.gov.sa/en/CoronaNew/P...09-07-001.aspx
HCP-MERS	Saudi Arabia	Confirmed	40	F	HCP	Unknown	Yes	Deceased	Unknown	http://www.moh.gov.sa/en/CCC/PressRe...02-28-001.aspx
HCP-MERS	Saudi Arabia	Confirmed	54	M	HCP	Unknown	Yes	Deceased	1/14/2014	http://www.peninsulatimes.org/2014/0...gladeshi-mers/
HCP-MERS	Saudi Arabia	Confirmed	26	M	Nurse	ER	No	Deceased	4/5/2014	http://www.aleqt.com/2014/03/28/article_836865.html
HCP-MERS	UAE	Confirmed	45	M	HCP	Unknown	No	Deceased	4/10/2014	http://www.who.int/csr/don/2014_04_14_mers/en/
HCP-MERS	Jordan	Confirmed	56	M	HCP	Unknown	Yes	Deceased	5/6/2014	http://www.assabeel.net/important/it...88%D9%86%D8%A7
HCP-MERS	Saudi Arabia	Probable	67	M	Doctor (Cardiologist)	Unknown	Yes	Deceased	9/5/2013	xhttp://www.alsharq.net.sa/2013/06/16/869245
HCP-MERS	Saudi Arabia	Confirmed	60	M	Dermatologist	Unknown	No	Deceased	5/12/2014	http://www.saudigazette.com.sa/index.cfm?method=home.regcon&contentid=20140514205096
HCP-MERS	Saudi Arabia	Confirmed	54	M	Home care Doctor	Private hospital	No	Deceased	5/12/2014	http://www.saudigazette.com.sa/index.cfm?method=home.regcon&contentid=20140514205096
HCP-SARS	Taiwan	Confirmed	52	F	HCW	Unknown	Yes	Deceased	4/19/2004	Provided by Taiwan University
HCP-SARS	Taiwan	Confirmed	42	M	Laundry staff	Laundry room	Yes	Deceased	4/29/2003	Provided by Taiwan University
HCP-SARS	Taiwan	Confirmed	37	F	Administration	Internal ward	No	Deceased	5/28/2003	Provided by Taiwan University
HCP-SARS	Taiwan	Confirmed	47	F	Nurse	Unknown	No	Deceased	5/1/2003	Provided by Taiwan University
HCP-SARS	Taiwan	Confirmed	28	M	Doctor	Internal ward	Yes	Deceased	5/15/2003	Provided by Taiwan University
HCP-SARS	Taiwan	Confirmed	41	F	Microbiologist	Testing Lab	Yes	Deceased	6/12/2003	Provided by Taiwan University
HCP-SARS	Taiwan	Confirmed	57	M	Care giver	Internal ward	Yes	Deceased	4/29/2003	Provided by Taiwan University
HCP-SARS	Taiwan	Confirmed	58	M	Care giver	Internal ward	No	Deceased	5/4/2003	Provided by Taiwan University
HCP-SARS	Taiwan	Confirmed	57	F	Cleaner	Cleaning room	Yes	Deceased	5/3/2003	Provided by Taiwan University
HCP-SARS	Taiwan	Confirmed	49	F	Nurse	Unknown	Yes	Deceased	5/18/2003	Provided by Taiwan University
HCP-SARS	Taiwan	Confirmed	59	M	Cleaner	Cleaning room	Yes	Deceased	5/27/2003	Provided by Taiwan University
HCP-SARS	Taiwan	Confirmed	51	F	Care giver	Ward	No	Deceased	5/23/2003	Provided by Taiwan University
HCP-SARS	Taiwan	Confirmed	68	F	Care giver	Ward	Yes	Deceased	7/16/2003	Provided by Taiwan University

**Denotations:** ER = Emergency room; HCP = Healthcare Personnel

## Discussion

MERS-CoV and SARS-CoV infections are caused by the β group of the Coronaviruses (The genomic size of coronaviruses ranges from approximately 26 to 32 kilobases, the largest for an RNA virus), but the two Coronaviruses have different sources of infection and evolution, as shown by phylogenetic studies [2, 12]. One unique common epidemiological characteristic of these two diseases is that the spread of both MERS-CoV and SARS-CoV infection has been largely driven by hospital-associated human-to-human transmission where HCP are affected the most. In addition, these two emerging infections displayed four major differences. First, the geographical distributions for the two infections were different, in that MERS cases occurred mostly in the Middle East areas and South Korea, but a significantly higher number of SARS cases happened in Asia, predominantly in Chinese-concentrated countries and areas, including China. The differing patterns of cases between MERS and SARS may represent varying ecological situations, animal-human contact behavior, surveillance, control efforts, and cross-country spread, etc. [7]. A recent imported MERS case from Korea to Guangdong, China resulted in zero cases in China, further supporting the public health significance of surveillance and contact tracing for emerging infectious diseases. As the spread of MERS has continued, additional countries and areas are anticipated to have future outbreaks due to unexpected new imported cases [13]. Secondly, the two diseases vary in the transmissibility and effectiveness of containment. SARS-CoV cases spread rapidly through person-to-person transmission, aided by super spreaders, and finally reached 8096 total cases and affected 29 countries worldwide in an 8-month period [14]. However, the MERS-CoV cases spread at a slower speed, resulting in less of an emergency than SARS, with a total of at least 1382 cases (as of 11 August 2015), and attacked 26 countries worldwide in a period of over 39 months, without successful containment [15]. However, the South Korean outbreak in hospital involved MERS coronaviruses, with early super-spreading events (29 cases) generating a disproportionately large number of secondary infections, and the transmission potential diminishing greatly in subsequent generations [16]. Aron J. et al. reported 48 health care worker contacts with one death with MERS-CoV, and showed no evidence of MERS-CoV infection, which suggested the transmission was limited, without sustained transmission [12]. Thirdly, global MERS cases had a CFR of 35.6%, much higher than the 9.6% in global SARS cases [17]. This high CFR was probably because many mild and even asymptomatic infections have not been identified, due to insufficient public health resources in the Middle East [18]. The actual CRF for MERS is most likely lower than the currently documented rate. However, the CFR varied for different areas, countries and ages. The CFR was 21% among overall MERS cases sourced from health settings[7]. CFR among patients aged 60 years or older under treatment was estimated at 48.2%, while the CFR among other cases was estimated to lie below 15%.The risk of MERS death was significantly associated with older age as well as treatment for underlying diseases, etc. [17]. Fourthly, for the MERS cases, 72% (118/163) were found in Saudi Arabia, where the mean age showed its predominance in older males, in contrast to SARS casesin the Taiwan. A gender difference was found in the different age subgroups among two groups. The differences between MERS-HCP and SARS-HCP did not contribute to the age differences between the HCP populations in Saudi Arabia and Taiwan, because the HCP distribution was similar in the two countries. The reasons are not fully understood, but have been attributed to cultural practices in which older men may be more likely to come in contact with camels and other MERS-CoV-positive animals, and male HCP may be more common in the healthcare settings in the Middle East [19].

HCP are the population at highest risk of getting the infection and transmitting the two respiratory CoV due to occupational exposure, and thus are affected mostly in nosocomial outbreaks. The outbreak of SARS in 2003 tragically highlighted a high CFR in HCP [5]. HCP with exposure to MERS-CoV also were found as a personal and family health risks and social stigmatization. Further epidemiological analyses found that younger nurses were the most affected for both diseases compared to total cases. In contrast, MERS-HCP cases were different from SARS-HCP cases, as shown by: (1) a lower percentage of total MERS cases from this study (13.37% vs 21.07%) [supported by the lower seroprevalence of HCP-MERS-CoV [12]]; (2) younger age and more male nurses (probably due to either occupational exposure or a cultural relationship [20]; (3) HCP acquiring the MERS-CoV mostly in ICU (compared to ER for most of the SARS-CoV cases) consistent with other reports [21]. Park BJ, et al. also reported that HCP exposure occurred most frequently in the emergency department (69%) and among nurses (47%) [22]. In other words, HCP with high-risk occupational exposure and working in high-risk areas of medical institutions should be well-educated, alerted, and tested once they demonstrate respiratory-related symptoms/signs, as in Toronto’s experience [23].

The clinical course of MERS generally follows a pattern typical of SARS infections. Our research estimated the incubation periods were 5~7 days in MERS-HCP and SARS-HCP groups, as documented from other reports [24]. Differently, the days from onset to confirmation in MERS-HCP were much shorter than for the total MERS cases. This is attributed to the fact that most HCP hade close contact with the primary case and under medical observation. Importantly, the clinical period (from onset to outcome) was much shorter in MERS-HCP than in SARS-HCP. This shows that MERS infections were much more severe, due to higher respiratory tract viral loads, than SARS infections [25]. In addition, this difference was related not only to detection capability, and earliness of medical seeking behavior, but also to medical care handling.

Coronavirus infections range from asymptomatic infection to severe pneumonia and even death. However, there are several important differences between both infections [17]. Firstly, the incidence of asymptomatic and mild infection remains 0.1% for the general population, and there are higher rates for HCP with SARS-CoV (0.22%, n = 453) in Beijing, China [26], far lower than that of MERS infections overall and in HCP respectively. As most MERS-HCP infections were identified in secondary cases, mainly as mild disease or asymptomatic infection [8], those MERS-HCP remained a potential infection source of MERS-CoV. Thus, there is an urgent need for developing a rapid, sensitive, and specific diagnostic test to confirm MERS-CoV infection [27]. Secondly, 70% (7/10) of fatal MERS-HCP were identified in male workers, while 61.5% (8/13) of fatal SARS-HCP were in female workers. However, the deadly cases were most often found in those in their 50s age groups with underlying diseases in both the MERS-HCP and SARS-HCP groups respectively. This sex distribution was attributed to gender occupational exposure, sanitation behaviors and gender-specific comorbidities. As for similarities, CFR for both HCP infections were much lower than that of overall infections. This might mean that MERS-HCP and SARS-HCP were due to early confirmation, as well as due to the younger age with fewer accompanying comorbidities and professional guidance in HCP groups. Another common point was that the fatal MERS-HCP and SARS-HCP cases were both associated with older age with comorbidities (e.g., diabetes, hematological diseases, smoking, etc.), as recorded in other reports [28].

The experience from controlling SARS in Asia was that nosocomial outbreak was associated with a lack of awareness of and preparedness for emerging infections. So, to prevent disease transmission to HCP, early and rapid detection of patients suspected of being infected with MERS SARS diseases would be beneficial to reduce the HCP and patient infections [13]. The second group of risks were overcrowding, medical operations generating virus-containing aerosols, and poorly ventilated environments. Another risk was substandard infection control practices. Therefore, future effort should include more comprehensive ways to control MERS-HCP infections. Firstly, pubic heath effort needs to emphasize establishing systems for early recognition and reporting of HCP cases through a computer-based syndrome surveillance system [29]. These vigilant virologic and epidemiologic surveillance for MERS and SARS viruses should be conducted in HCP in nationwide in winter and spring of every year, which will be valuable to decrease the incidence, severity and transmission of two viruses in HCP working in health settings. Secondly, environmental control should be implemented through better engineering design, ventilation and disinfection in hospitals, regular monitoring and check-up and management policies [30]. Thirdly, rigorous training and practice of the standard operating infection-control procedures, including isolation of suspected or confirmed MERS-CoV cases, putting on and taking off personal protective equipment (PPE) and evaluations thereafter, are necessary.

### Ethical Considerations

The MERS data were obtained from publicly available data sources. Since SARS was a legally required notifiable disease in Taiwan in 2003, the data of SARS cases were centralized as a data bank for group data analysis only. All these data were provided and analyzed in an anonymous format, without access to personally identifying information.

## Supporting Information

S1 Table Summary of MERS-HCP cases with onset of illness from April 1 of 2012 to July 7 of 2015. (XLSX)

S2 Table Summary of SARS-HCP cases with onset of illness from November 1 of 2002 to December 31, 2003. (XLSX)

## Abbreviations

ARDS: Acute respiratory distress syndrome
CFR: Case-fatality rate
ELISA: Enzyme-linked immunosorbent assay
ER: Emergency room
HCP: Healthcare Personnel
HH: Households
ICU: Intensive care unit
IFA: Immunofluorescent antibody
ILI: Influenza-like illness
MERS: Middle East Respiratory Syndrome
MERS-CoV: Middle East Respiratory Syndrome Coronavirus
PPE: Personal protective equipment
rRT-PCR: Reverse transcription polymerase chain reaction
SARS: Severe Acute Respiratory Syndrome
SARS-CoV: Severe Acute Respiratory Syndrome Coronavirus
SOB: Shortness of breath
